# Comparison Between Modified Lateral Arm Free Flap and Traditional Lateral Arm Free Flap for the Reconstruction of Oral and Maxillofacial Soft Tissue Defects

**DOI:** 10.3389/fonc.2022.877799

**Published:** 2022-05-26

**Authors:** Wei-Ming Wang, Lu Sun, Si-Si Yang, Shu-Jun Hu, Yi-Jie Zuo, An-Jie Min

**Affiliations:** ^1^Department of Oral and Maxillofacial Surgery, Center of Stomatology, Xiangya Hospital, Central South University, Changsha, China; ^2^National Clinical Research Center for Geriatric Disorders, Xiangya Hospital, Changsha, China; ^3^Institute of Oral Precancerous Lesions, Central South University, Changsha, China; ^4^Research Center of Oral and Maxillofacial Tumor, Xiangya Hospital, Central South University, Changsha, China

**Keywords:** modified lateral arm free flap, leaf flaps, chimeric flaps, propeller flaps, oral squamous cell carcinoma

## Abstract

**Objective:**

The traditional lateral arm free flap (tLAFF) has the disadvantages of short vascular pedicle, small vascular diameter, and non-perforator flap. We used a new method to prepare modified LAFF (mLAFF) and evaluate its application value in the repair of oral and maxillofacial soft tissue defects.

**Methods:**

The anatomical features of the flap were recorded and compared between the tLAFF group and the mLAFF group. All the flaps in the modified group were perforator flaps. Statistical analysis was performed on the data using ANOVA on SPSS 22.0 statistical software package.

**Results:**

Forty-five mLAFFs were prepared as eccentric design rotation repair perforated flap, or multi-lobed or chimeric perforator flaps. Compared with the tLAFF, the vascular pedicle length of the mLAFF was increased, and the outer diameter of the anastomosis was thickened. The damage to the donor site was less. The difference was statistically significant.

**Conclusion:**

The mLAFF can effectively lengthen the vascular pedicle length and increase the anastomosis diameter. Perforator LAFFs in the repair of oral and maxillofacial defects have good application value.

## Introduction

Soft tissue defects caused by tumors and trauma in the oral and maxillofacial region require repair and reconstruction by transplanting autologous tissue from other parts of the body (1). In recent years, the lateral arm free flap (LAFF) has been used to repair the skin, limbs, penis, and oral and maxillofacial soft tissue defects (2–4). However, the flap has shortcomings, such as short and thin vascular pedicle, limiting its wide application (2, 5).

This study investigates the use of perforator flaps in the reconstruction of the head, neck, and limbs. The perforator flaps improve the shape and function of the flap recipient site, reduce the appearance and functional damage of the donor site, and flexibly transfer the flap (6, 7). The appearance of perforator flaps has led to the development of various forms of flaps, such as chimeric and multi-lobed flaps (8). Previous studies showed that the LAFF is taken together with the muscles and fascia of the deep vascular pedicle (2, 5). Therefore, the flap is unsuitable for the repair of certain oral and maxillofacial defects.

In this study, we modified the preparation method of the flap based on the clinical anatomical research of the position of the perforators and developed novel styles of this flap. The characteristics of the modified flaps were compared with those of the traditional flaps.

## Materials and Methods

### Normal Information

The present study was approved by the Medical Ethics Committee of Xiangya Hospital, Central South University (Hunan, China) and was performed according to the Declaration of Helsinki guidelines on experimentation involving human subjects. All patients signed informed consent. Sixty-two patients who underwent LAFF repair at the Oral and Maxillofacial Surgery Department of Xiangya Hospital from March 2012 to April 2017 were enrolled in this study. The specific information of patients is shown in **Table 1**.

**Table 1:** Demographics, recipient site, and lesion size of lesions undergoing LAFF reconstruction.

	tLAFF	mLAFF
Age (mean years)	50.82	45.45
Sex		
Male	16	41
Female	1	4
Recipient site		
Tongue	7	18
Cheek	3	10
Gingiva	4	9
Floor of mouth	2	5
Oropharynx	1	3
T stage		
T1	5	16
T2	10	23
T3	2	6
N stage		
N0	10	26
N1	7	19
No. of total flaps	17	69

### Preoperative Positioning of Perforating Vessels

Preoperative exploration of the perforator of the LAFF was performed through color Doppler Ultrasound (CDUS) (PHILIPS Epic5). The dominant vessel with the largest diameter and the highest blood flow peak was selected and designated as the first perforating branch, and the distance from the stop point of the deltoid muscle was measured.

### Surgical Methods

The design and cutting of LAFF of the traditional group are consistent with those described in the literature. Additional details for the specific methods can be found in the **Supplementary Method**.

### Design and Preparation of mLAFF

#### Improvement 1 (Improvement of Surgical Incision and Vascular Pedicle Preparation)

**Figure 1:**
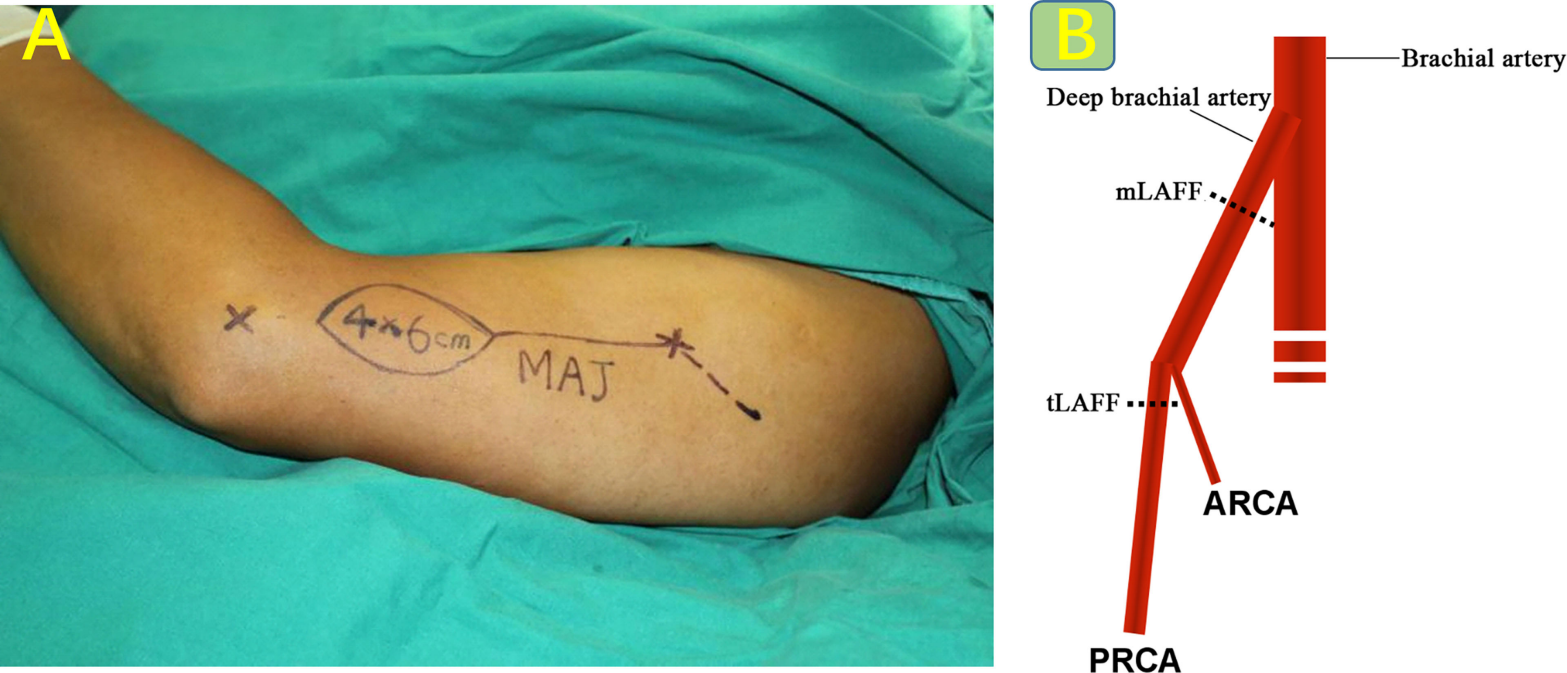
**(A)** Improvement of surgical incision, as shown in the dotted line. **(B)** Schematic diagram of preparation of modified vascular pedicle.

The lower end of the flap is located 3 cm to 6 cm above the lateral epicondyle of the humerus to avoid the elbow joint. The upper incision is located 3 cm to 6 cm posteriorly along the outer edge of the deltoid muscle at the deltoid stop to better expose the deep brachial artery. The upper segment of the deep brachial artery was divided to obtain an ideal vascular pedicle length and caliber (**Figure 1**).

#### Improvement 2 (Special Form of LAFF: Eccentric Design, Rotation Repair Perforated Flap Is Abbreviated as EDR-LAFF in the Following Text)

**Figure 2:**
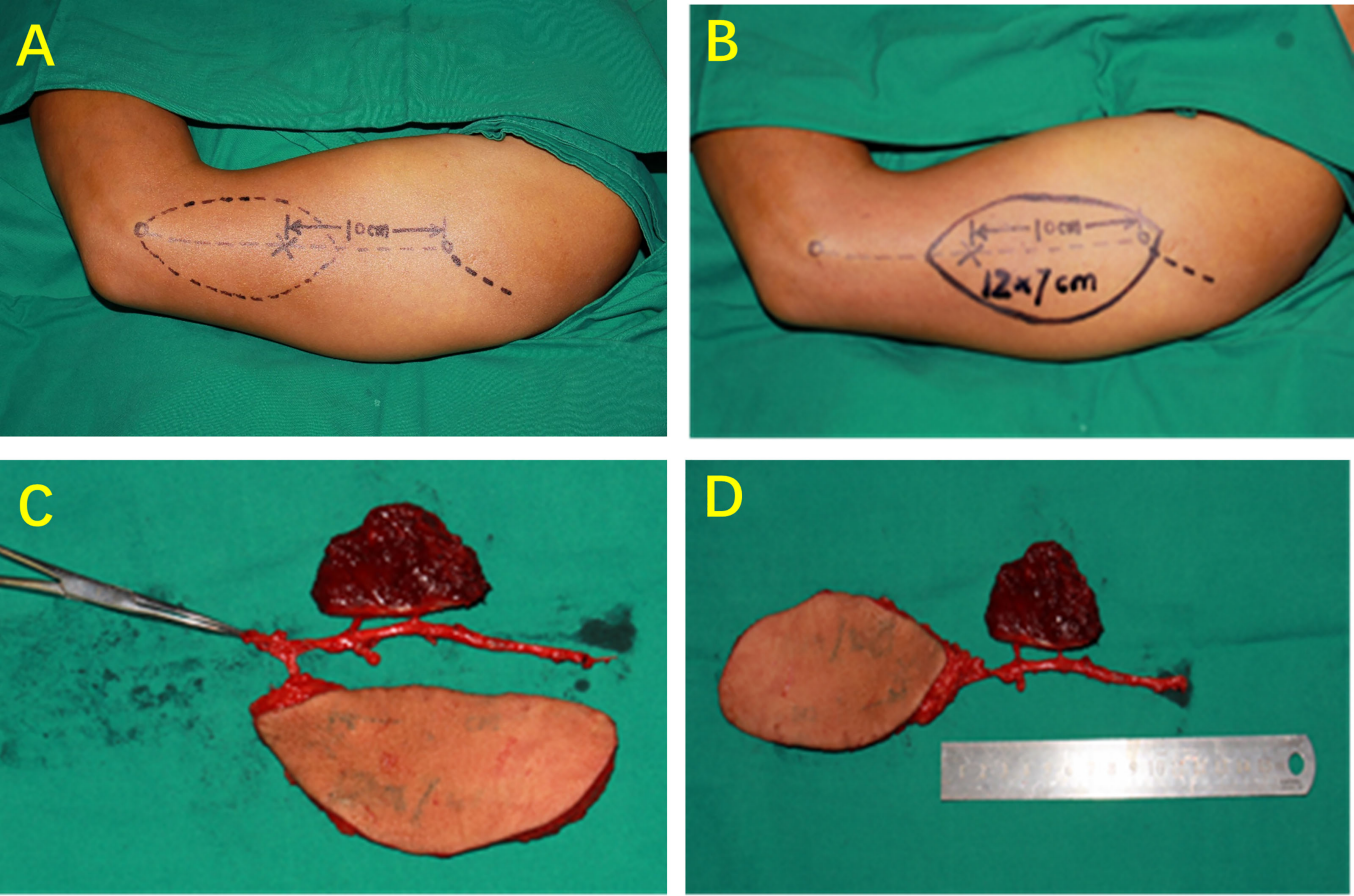
Eccentric design, rotation repair perforated flap. **(A)** Schematic of traditional skin island design. **(B)** Schematic of eccentric design, rotation repair perforated flap design. **(C)** Before rotation, the pedicle length was 10 cm. **(D)** After rotation, the total length of the flap and vascular pedicle reached 24 cm.

For this type of flap, the perforator is designed at the proximal end of the flap, and the skin island is rotated 180° during repair and reconstruction. The purpose of this design is to shorten the distance between the recipient vessel and the flap pedicle. This design extends the distance between the distal end of the flap and the recipient vessel. The perforator vessel was positioned by CDUS before operation, and a single reliable perforator point was used as the rotation point of the perforator flap, and it was intentionally designed to the eccentric side of the lower end of the flap. Through the fine dissection and release of a single perforating vessel, it can obtain a certain range of motion. After the flap is prepared, rotate 180° with the perforator as the rotation point, so that the proximal end of the donor site of the flap becomes the distal end of the repair site (**Figure 2**).

#### Improvement 3 (Special Form of LAFF: Multi-Lobed + Chimeric Combined Flaps)

**Figure 3:**
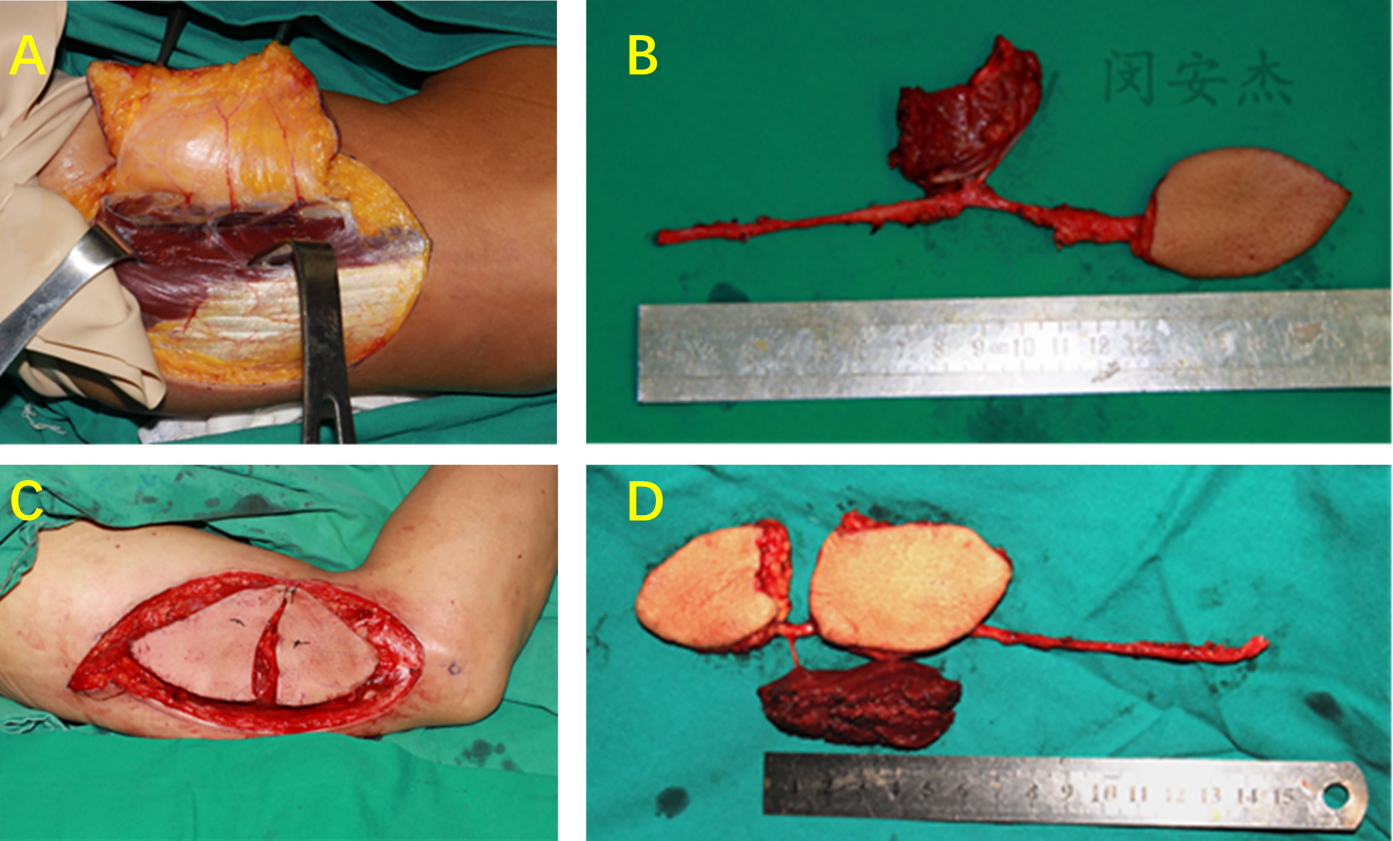
Multi-lobed + chimeric combined flap. **(A)** Dissection of PRCA skin perforator and muscle perforator. **(B)** Chimeric LAFF. **(C, D)** Multi-lobed + chimeric combined flap.

For more complex soft tissue defects involving multiple anatomical regions, a multi-lobed or/and chimeric LAFF can be prepared as a skin island-muscle flap. The perforator vessels were located by CDUS before operation, and the multi-lobed type LAFF was designed and prepared by supplying blood from different skin perforator vessels respectively. If necessary, the muscle perforator can be used as a pedicle to cut part of the triceps muscle flap. Chimeric flap has separate components with separate vascular supplies that are attached to a common vascular pedicle; its components may comprise either similar or different tissues, such as skin, muscle, and bone. The multi-lobed free flap refers to the separation of multiple independent flaps from the same main vessel, and each flap has an independent perforator blood supply. These two special forms of perforator flaps are anastomosed with a set of vascular pedicles to ensure the survival of more than two tissue flaps (**Figure 3**).

### Intraoperative Data Measurement

The outer diameter of the vascular pedicle, the tissue thickness of the flap, the length of the vascular pedicle, the length and width of the flap, and the distance from the first perforator to the deltoid stop point were measured intraoperatively.

### Postoperative Data Collection and Efficacy Evaluation

The survival rate of the flap at the time of discharge was recorded. The upper limb two-point discrimination and overall function evaluation of the elbow joint (HSS, Heidke Skill Score) were recorded. Additional details for the specific methods can be found in the **Supplementary Method**.

### Statistical Analysis

In this study, SPSS 22.0 was used to statistically analyze the data, and the measurement data were tested in terms of normality and variance homogeneity. Statistical description: the measurement data conforming to the normal distribution are expressed as $\left(\bar{X}\pm S\right)$ , and the count data are represented by *n* (%) or score. Table scores are indicated. Statistical inference: *t*-test was used to compare the measurement data between the two groups, and *χ^2^* test was used to compare the count data. The test level was *α* = 0.05.

## Results

### Vascular Pedicle and Perforation Distribution Characteristics

Two to six skin perforating vessels and several muscle branches are found in the middle and lower arms of the upper arm in all patients through CDUS detection and intraoperative verification. All skin perforators are intermuscular perforators. The distance between the first perforating distance and the deltoid point is 10.4 ± 0.95 cm (9.3 cm to 13 cm), and the anatomical position is relatively constant.

### Flap Type and Survival Rate

The modified group included 29 multi-lobed or chimeric flaps and 16 EDR-LAFFs, with a total of 69 free tissue flaps, including 48 flaps and 21 muscle flaps. One case of skin flap necrosis was found in the modified and traditional groups, and no significant difference was observed in the rate of skin flap necrosis (**Supplementary Table 1**) (*p >* 0.05).

### General Characteristics of the Flap

The flap area, vascular pedicle length, and vascular pedicle arteriovenous diameter of the modified group were statistically larger than those of the traditional group (*p <* 0.05). The thickness of the flap and the incidence of numbness in the donor site were lower than those of the traditional group, and the difference was statistically significant (*p <* 0.05) (**Table 2**).

**Table 2:** General characteristics of the flap.

	tLAFF	mLAFF	*p*-value
Vascular pedicle length, cm	9.29 ± 0.83	12.08 ± 1.89	0
Artery diameter, mm	0.92 ± 0.04	1.64 ± 0.17	0
Vein diameter, mm	1.15 ± 0.13	2.37 ± 0.23	0.027
Flap breadth, cm	5.23 ± 0.81	7.53 ± 1.72	0.012
Flap length, cm	7.70 ± 1.68	12.38 ± 2.95	0.017
Flap thickness, mm	8.16 ± 0.36	4.25 ± 0.38	0

### Two-Point Discrimination Distance in the Donor Site

No significant difference was found in the two-point discrimination distance between the upper arm lateral skin and the posterior lateral forearm skin in the modified group (*p >* 0.05). A statistical difference was observed between the traditional group (*p <* 0.05) (**Table 3**).

**Table 3:** Two-point discrimination distance in the donor site.

	Pre-operation (mm)	12-month postoperation (mm)	*p*-value
Upper arm lateral skin			
tLAFF	42.92 ± 2.01	66.39 ± 5.61	0
mLAFF	42.84 ± 2.60	47.34 ± 3.30	0.116
Posterior lateral forearm skin			
tLAFF	33.6 ± 0.58	67.98 ± 1.62	0
mLAFF	33.43 ± 2.08	36.79 ± 2.75	0.069

### Elbow Joint Motor Function Assessment and Overall Elbow Joint Status Score (Heidke Skill Score)

The flexion, supination, and supination angles of the traditional and modified groups were not significantly different between the 6th and 12th months after surgery (*p >* 0.05). The flexion elbow angle of the traditional group at 6 and 12 months after surgery was significantly greater than that before surgery (*p <* 0.05). No significant difference was observed between the modified and preoperative groups (*p >* 0.05). In the modified group, the overall state of the elbow joint was superior to the traditional group, and the average score of HSS was statistically different (**Tables 4**, **5**).

**Table 4:** Elbow joint motor function assessment of the traditional groups.

	Pre-operation	6 months postoperation (°)	12 months postoperation (°)	*p*-value
Elbow bending	142.8 ± 3.54	128.9 ± 4.10	132.2 ± 4.16	0
Elbow extension	0.47 ± 0.79	0.64 ± 0.93	0.41 ± 0.71	0.843
Pronation	85.29 ± 2.51	82.76 ± 1.92	82.29 ± 1.49	0.053
Supination	84.53 ± 2.21	81.94 ± 1.78	81.24 ± 1.95	0.066

**Table 5:** Elbow joint motor function assessment of modified groups.

	Pre-operation	6 months postoperation (°)	12 months postoperation (°)	*p*-value
Elbow bending	142.5 ± 4.52	139.57 ± 3.63	140.29 ± 3.24	0.852
Elbow extension	0.62 ± 0.86	0.51 ± 1.15	0.55 ± 1.59	0.509
Pronation	85.11 ± 2.47	80.98 ± 3.03	82.42 ± 2.65	0.15
Supination	84.51 ± 2.81	80.47 ± 2.45	81.84 ± 1.96	0.125

### Typical Cases

#### Case 1 (EDR-LAFF)

**Figure 4:**
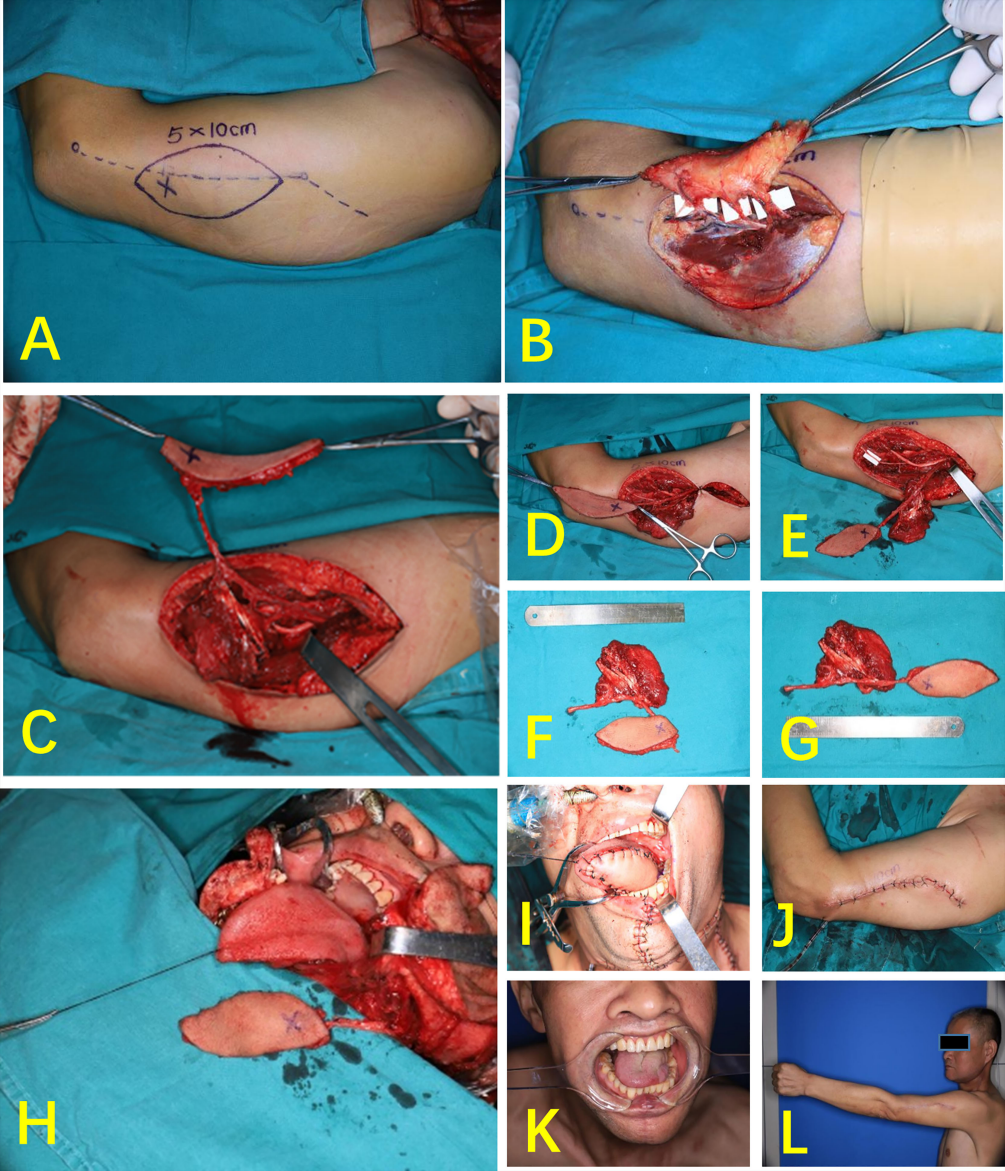
Case 1 (EDR-LAFF). **(A)** Flap design. **(B)** Three cutaneous perforators were found during the operation. **(C)** The perforator of the distal end was retained. **(D)** The flap was rotated by 180°. **(E)** Chimeric flap (the white paper shows posterior cutaneous nerve of the arm). **(F)** The length of the pedicle before rotation was 9 cm. **(G)** The total length of the flap and pedicle after rotation was 18 cm. **(G)** Chimeric combined flap. **(H)** Vascular anastomosis. **(I)** Repair of defects intraorally. **(J)** Donor side after suturing. **(K)** Skin island of the flap intraorally, 1 month postoperatively. **(L)** Elbow extension, 1 month after surgery.

A 49-year-old male patient was admitted to the hospital because of “right tongue mass with pain for more than 1 month”. The patient was diagnosed with right tongue squamous cell carcinoma (cT2N0M0). A modified left LAFF of 5 cm × 10 cm was designed in accordance with the defect of the recipient. Three perforating branches were found during the operation, but the two perforating vessels near the proximal end intersect the lateral cutaneous nerves of the arm. The flap blood supply was normal after blocking the two perforating vessels near the heart end for 0.5 h. The proximal end of the two perforating vessels was ligated, leaving only one perforating vessel at the distal end. The flap was rotated by 180°, and the overall length of the flap and the vascular pedicle was extended from 9 cm to 18 cm. After the operation, the shape of the tongue was satisfactory, and the donor site was directly sutured. No restriction was found on the motor function of the elbow and wrist in the donor site, and no numbness was observed in the donor site (**Figure 4**).

#### Case 2 (Multi-Lobed + Chimeric Combined Flap)

**Figure 5:**
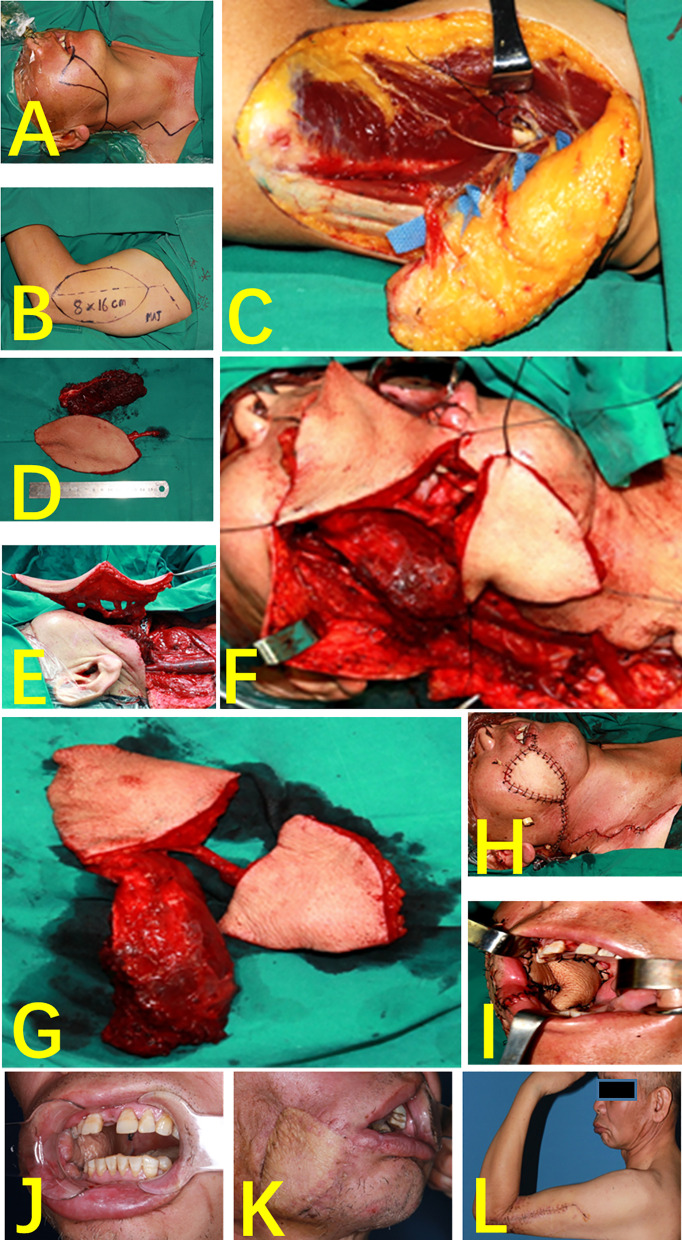
Case 2 (multi-lobed + chimeric combined flap). **(A)** Perforated resection of right buccal carcinoma. **(B)** Flap design. **(C)** Three cutaneous perforators and one muscle perforator were found during the operation. **(D)** Chimeric flap. **(E)** Vascular anastomosis. **(F)** The multi-lobed flap was designed according to the area of the defect size of inside and outside the mouth. **(G)** Multi-lobed + chimeric combined flap. **(H)** Repair of cheek defects. **(I)** Repair of buccal mucosa defects. **(J)** Skin island of the flap intraorally, 1 month postoperatively. **(K)** One month after surgery, right facial flap. **(L)** Elbow bending, 1 month after surgery.

A 52-year-old patient had a highly differentiated squamous cell carcinoma of the right cheek (cT3N0MO). The radical chelation of the right cheek cancer resulted in a cheek-transmissive defect and left a huge cavity in the masseter muscle area of the parotid gland. During the operation, the position of the three perforators was consistent with the location of the preoperative CDUS. In accordance with the position of the perforator flap and the defect size, the flap was designed as a two-lobed flap, and a part of the triceps was used to prepare a muscle flap. The two-lobed flap repairs the intraoral and extraoral defects, and the muscle flap fills the cavity of the parotid masseter muscle. All the three tissue flaps completely survived after operation. Compared with the preoperative phase, no difference was found in the sensory and motor functions of the donor site after 3 months (**Figure 5**).

## Discussion

The LAFF is a perforator flap with a posterior branch of the radial accessory artery as the vascular pedicle (9–11). The radial accessory artery is a terminal branch of the deep brachial artery and has an anatomical position. One to six skin branches were emitted from the radial accessory artery based on the microsurgical anatomy of specimens (2, 9). The outer diameter of the branch is 0.1–1.4 mm, and the probability of perforation greater than 0.8 mm is 29.1% (12). Kun Hwang conducted an anatomical study on the perforation of the upper arm using a cadaver specimen. The upper arm had four constant perforating branches, including one inner arm and three outer arms (13).

Two to six percutaneous branches and muscle perforators were found in all cases of the modified group through the microscopic anatomy of the perforating branch of the radial artery. The first perforator is constantly located at approximately 10.4 ± 0.95 cm from the deltoid stop. The relatively constant position of perforation is convenient for the preparation of the perforator flap.

The preparation method of the tLAFF has shortcomings, such as short vascular pedicle, thin blood vessel diameter, high requirements for anatomical flap, and high microscopy technique, thereby limiting its popularization and application (2, 5, 14). Kuek et al. designed an extended LAFF (ELAFF) that extends the lower end of the flap to the upper middle of the forearm to increase the vascular pedicle length (15). However, this method causes the scar to be evident and exposed, does not help to increase the vascular anastomosis diameter, and may cause dyskinesia of the elbow joint. We modified the location of the flap incision and the position of the vascular pedicle to increase the overall length and diameter of the vascular pedicle. The results show that the mLAFF can extend the average length of the vascular pedicle of the tLAFF by approximately 23.09%. Compared with the tLAFF and ELAFF, the length of the vascular pedicle is significantly increased, and the diameter of the modified vascular pedicle is increased to meet the requirements of microvascular anastomosis in oral and maxillofacial surgery. A special form of LAFF—a multi-lobed flap—can convert the flap width to length, allowing the donor site to be sutured and sutured directly. The muscle flap in the chimeric flap can fill the soft tissue cavity and reduce the risk of postoperative infection.

The sensory nerves associated with the LAFF are mainly the posterior cutaneous nerve of the arm (PCNA) and the forearm cutaneous nerve (PCNF) (16, 17). The PCNA and PCNF are accompanied by PRCA because they are located in the subcutaneous tissue layer and often intersect the vascular pedicle of the flap and the perforating vessel (12, 18). The tLAFF was not dissected to the perforating vessels and nerves, and the patient’s lateral upper arm and forearm medial skin were significantly numb. The mLAFF microscopically dissected the perforating vessels during preparation. A lobed flap with different skin perforating branches was prepared in accordance with the position of the perforating branch to relieve the nerve and the perforating branch. The perforating blood vessel was ligated to completely retain the cutaneous nerve if the perforating blood vessel crossing the nerve was a nondominant blood vessel. In the traditional group, the preoperative two-point discrimination distance of the patient’s upper arm lateral and forearm posterior superior skin was significantly greater than the two-point discrimination distance of 1 year after surgery. In the modified group, no significant difference was found in the two-point discrimination distance of the patient’s upper arm lateral and forearm posterior superior skin between presurgery and 1 year after surgery. The modified group has less damage to the sensory function of the donor site than the traditional group due to the protection of the PCNA and PCNF. The recovery of sensory sensitivity is better than that of the traditional group, and the damage to the sensory function of the donor site is smaller.

In summary, the mLAFF can effectively prolong the vascular pedicle length and increase the vascular pedicle diameter. Specialized perforating flaps with special forms, such as multi-lobed type, chimeric, and EDR-LAFF, are prepared according to the perforating branches’ location. The improvement based on the characteristics of the piercing branch expands the applicable range and repair effect of the flaps and reduces the influence on the feeling and movement function of the donor zone. However, the mLAFF has limited area and cannot repair large areas of soft tissue defects, which needs further research and improvement.

## Data Availability Statement

The original contributions presented in the study are included in the article/**Supplementary Material**. Further inquiries can be directed to the corresponding author.

## Ethics Statement

The studies involving human participants were reviewed and approved by the Medical Ethics Committee of Xiangya Hospital, Central South University (Hunan, China). The patients/participants provided their written informed consent to participate in this study.

## Author Contributions

W-MW and LS: concept/design, data analysis, and surgical team members. S-SY, S-JH, and Y-JZ: data analysis and surgical team members. A-JM: critical revision, final approval, and surgical team lead. All authors contributed to the article and approved the submitted version.

## Funding

This study was funded by the National Natural Science Foundation of China (grant no. 81702708) and the Natural Science Foundation of Hunan (grant no. 2018JJ3862).

## Conflict of Interest

The authors declare that the research was conducted in the absence of any commercial or financial relationships that could be construed as a potential conflict of interest.

## Publisher’s Note

All claims expressed in this article are solely those of the authors and do not necessarily represent those of their affiliated organizations, or those of the publisher, the editors and the reviewers. Any product that may be evaluated in this article, or claim that may be made by its manufacturer, is not guaranteed or endorsed by the publisher.
