# Supplementary material for: Comparison Between Modified Lateral Arm Free Flap and Traditional Lateral Arm Free Flap for the Reconstruction of Oral and Maxillofacial Soft Tissue Defects
Source: Front Oncol. 2022 May 26;12:877799. doi: 10.3389/fonc.2022.877799 (PMC9178180; doi:10.3389/fonc.2022.877799)
Supplement: Supplementary file 1 [file DataSheet_1.docx]

**Supplementary**

Supplementary method

Traditional LAFF harvesting operation

A line was drawn between the deltoid insertion point and lateral epicondyle of the humerus, and the flap was centred over the line. The flap skin paddle marking was drawn. First, a posterior margin incision was made to the skin and subcutaneous fatty tissues down to the branchial fascia. Subfascial dissection was performed continuing anteriorly until the posterior intermuscular septum was reached. Next, the anterior flap outline was incised down to the brachialis and brachioradialis fascia. The pedicle was then identified from the lateral intermuscular septum. When the vascular pedicle was seen in its entirety from its anterior aspect, the septum was isolated from the periosteum of the humerus from the distal to the proximal. The vascular pedicle length, vascular diameter, and the thickness at the proximal, middle, and distal parts of the anterior margin of the flap were measured.

Two-point discrimination test

Two-point discrimination is a quantitative index to measure the sensory function of the skin. It can be used to evaluate the sensitivity of the skin sensation in the operation area, and to judge the patient's sensory nerve damage and recovery. It is usually performed 6 and 12 months after the surgery. On the premise that the patient's eyes are closed, the doctor stimulates the skin with separate foot gauges, and pays attention to avoid puncturing the patient (the skin at the point where the needle tip is pressed is slightly white). The distance between the two points is generally carried out in descending order. When the patient can feel two points, repeatedly shorten the gauge distance (about 1mm each time) until the patient feels one point. At this time, the distance of the gauge is the measured two-point discrimination distance. The smaller the distance, the more sensitive the patient's skin is to external stimuli.

HSS score

The HSS score is a scale commonly used to study elbow function. On the basis of measuring the angles of elbow flexion, extension, pronation and supination, the patient's donor site appearance, scar, sensation, muscle strength, and patient satisfaction were scored using the elbow joint HSS scale.

S table 1 Flap survival rate

|  | survival | necrosis | p value |
| --- | --- | --- | --- |
| tLAF | 16 | 1 |  |
| mLAF | 69 | 1 | 0.3545 |
